# Generative AI for predictive breeding: hopes and caveats

**DOI:** 10.1007/s00122-025-04942-8

**Published:** 2025-06-11

**Authors:** M. Pérez-Enciso, L. M. Zingaretti, G. de los Campos

**Affiliations:** 1 Centre for Research in Agricultural Genomics (CRAG), CSIC-IRTA-UAB-UB, Campus UAB, 08193 Bellaterra, Barcelona, Spain; 2 Institució Catalana de Recerca I Estudis Avançats (ICREA), 08010 Barcelona, Spain; 3 Departments of Epidemiology and Biostatistics, Michigan State University, East Lansing, MI 48824 USA; 4 Institute for Quantitative Health Sciences and Engineering, Michigan State University, Michigan State University, East Lansing, MI 48824 USA; 5 Department of Statistics and Probability, Michigan State University, East Lansing, MI 48824 USA

## Abstract

Among the broad area of artificial intelligence (AI), generative AI algorithms have emerged as a revolutionary technology able to produce highly realistic ‘synthetic’ data, akin to standard simulation but with fewer contraints. The main focus of generative AI has been on phenotypes, but here we argue it can serve as well for generating synthetic environments and genotypes. This data-driven technology may be able to overcome some of the limitations that standard simulations have, such as strong assumptions on the underlying genotype to phenotype map. We discuss key features of popular generative models including autoregressive models, generative adversarial networks, variational autoencoders, diffusion and flow-based models. Several of these methods utilize a latent space, often of lower dimensionality than the raw data, that can help making the models interpretable and can be a link between simulation and generative algorithms. Augmenting data as realistically as possible with genAI can improve inference and predictive performance of genomic prediction models, but symbolic simulation will continue to play a fundamental role in predictive breeding. A hybrid tool that implements both approaches can be extremely powerful to evaluate predictive breeding strategies in silico. One promising direction is to simulate novel genotypes using conventional methods, then apply generative models to produce realistic phenotypes conditional on genotype and environment.

**Supplementary Information:**

The online version contains supplementary material available at 10.1007/s00122-025-04942-8.

## Introduction

Plant and animal breeding has been traditionally based on mixed model theory and the infinitesimal model. Although their underlying principles are extremely robust, not all questions of interest can be answered using theory only. There are no closed formulae to predict selection response allowing for genetic drift, to optimize complex breeding schemes or to predict loss of genetic diversity in a generic scenario, to name but a few questions. Computer simulation, in turn, can be easily applied to solve these and many other issues, saving expensive experimental resources. For those reasons, computer simulation has been a fundamental tool in plant and animal breeding for decades (Li et al. 2012; Bančič et al. 2025; Hassanpour et al. 2025).

Usually, simulations in plant and animal breeding have been done using a ‘symbolic’ approach: a computer code prescribes how ‘nature works’, including how alleles segregate at meiosis, what loci affect phenotypes and how these alleles act, what processes govern mating and selection decisions and how the environment and genetics mediate performance, e.g., via crop growth models (Asseng et al. 2014). The simulation is then run to elucidate properties of the system under the desired scenarios.

Standard simulations may have some limitations though. For instance, there is significant ambiguity about how nature actually works, e.g., most causative loci and their effects are unknown. A second limitation of standard simulations is that they return phenotypes with well known distributional properties, such as a multivariate normal distribution. However, many traits of interest in plant or animal breeding do not have well defined statistical properties, or are intrinsically multidimensional. Examples are fruit shape, plant architecture and development, among many others.

In parallel, rapid developments in artificial intelligence (AI) are having a profound impact in Agriculture, including predictive breeding. AI applications range from remote sensing (Wang et al. 2022), disease detection (Pacal et al. 2024), genomic prediction (Pérez-Enciso and Zingaretti 2019; Crossa et al. 2025) to breeding optimization (Younis et al. 2024). Among the broad area of AI, generative AI algorithms (genAI for short, Goodfellow et al. 2016) have emerged as a revolutionary technology able to produce highly realistic ‘synthetic’ data, akin to simulation but with fewer contraints and much more general. Unlike traditional simulations, generative AI uses patterns learned from data to generate new data. For instance, a Language Model learns patterns from existing text that can be used for multiple tasks, including producing summaries in response to questions prompted to the system, guessing the next sequence of words, generating a new song or a new figure in response to a prompt. This technology may overcome some of the limitations that symbolic, standard genetic simulations have.

Symbolic simulations and generative approaches are based on different principles and can tackle the same or different tasks. In this article, we clarify the distinction between genAI and traditional simulation technologies, provide an overview of the most popular genAI algorithms, and discuss their advantages and limitations. We end by hypothesizing on future developments and ways to combine the best of both approaches. We focus on applications of genAI in predictive breeding to minimize overlap with other generic reviews in Agriculture (e.g., Pallottino et al. 2025). 

## Setting the stage

**Fig. 1:**
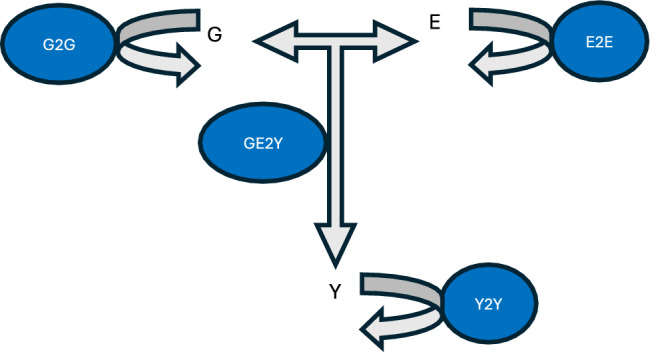
Graphical representation of a conceptual simulation platform that includes four modules: G2G, E2E, Y2Y and GE2Y, where **G**, **E** and **Y** refer to genotypes, environment and phenotypes, respectively. Module G2G generates progeny genotypes from parental or extant genotypes. Mating decisions could be based on information on the observed phenotypes and genotypes of ancestors (breeding and selection decisions) or could be generated by DNA language models. E2E simulates environmental data. This could be simulated data or observed historical weather data. Y2Y simulates new phenotypes based on extant phenotypes. Finally, module GE2Y uses genotypic and environmental information to simulate phenotypes. The GE2Y is usually the most complex module, and can incorporate a range of physiological layers to increase realism, at the cost of losing generality and ambiguity in the values of parameters. All these modules can be developed using a symbolic or data driven methods

There are three major variables of interest in a breeding program: the genotypes (**G**), the environment (**E**), and phenotypes (**Y)**. A complete simulation platform involves many steps that can be encapsulated in several ‘modules’ (Fig. 1): Simulating progeny genotypes from parental genotypes (G2G), simulating environmental conditions (E2E), and simulating phenotypes from genotypic and environmental information (GE2Y). Moreover, simulation can be replaced partially or totally by real data: instead of simulating environmental conditions, historical weather data could be used. Likewise, real genotypes can be used in lieu of simulated ones. For multi-generation simulations, selection and mating decisions are also part of the pipeline connecting one generation to the next. In standard breeding simulation schemes the simulation modules above-described are implemented using a symbolic approach which prescribes how nature works, e.g., how many loci affect a phenotypes, what are the effects of those loci, and whether and how alleles interact among them and with environmental conditions. For example, the map from environmental conditions to phenotypes, or even the entire GE2Y, may be represented using a crop growth model (Asseng et al. 2014; Messina et al. 2018). In this approach a computer algorithm is used to represent physiological knowledge about the map from environmental conditions and genotypes to phenotypes.

Each of the modules in Fig. 1 can potentially be implemented using genAI. For example, using genotypes of parents and offspring, one could fit a machine learning model (e.g., a transformer, Vaswani et al. 2017) to capture the patterns relating parental genotypes to offspring genotypes and the trained model could simulate new progeny genotypes when prompted with parental genotypes as inputs. Likewise, once could envisioning fitting an AI model using parental and offspring phenotypes and then using it in a generative AI fashion to simulate offspring phenotypes from parental phenotypes. An autoregressive E2E module could learn from past environmental data (temperature, precipitation) to generate new plausible conditions. Finally, using extensive trial data, including phenotypes, genotypes, and environmental variables, a general AI model could learn how genotypes react to environmental conditions and the resulting model could be used to generate new phenotypes from genotypes and environmental conditions generated by a G2G and E2E modules (de los Campos et al. 2020). We argue that generative AI is a promising avenue for building more powerful simulation platforms.

## The classical paradigm: symbolic simulation

In a symbolic simulation, a computer code represents the assumed nature mechanisms. Although simulation can be used to produce phenotypes, environments, or genotypes, here we focus on simulating genotypes, as it is the most widely used application. Module G2G specifies how genotypes of the progeny are simulated from the genotypes of the parents, a process that starts by sampling recombination events to generate new haplotypes and then mating. The computer code specifies how the process works, including points of uncertainty, e.g., sampling of recombination points and possible mutations. It is worth asking: What is the purpose of simulations if nature mechanisms are known? Normally, simulations are run to elucidate properties of the process that are not self evident, such as the effects of selection on linkage disequilibirum, or the expected response to selection decision rules. The output of the simulation can then be validated by comparing the distribution of the simulated data with that of observed data.

**Fig. 2:**
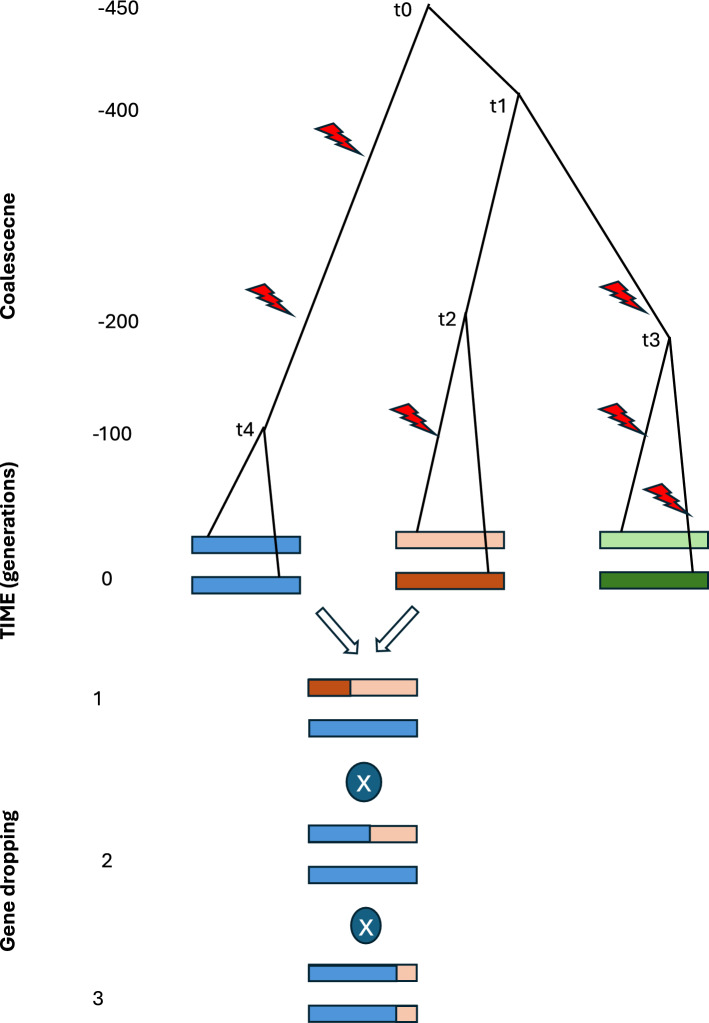
A graphical comparison between upward (coalescence) and forward (genetic dropping) genetic simulations. Figure represents an ancestral graph of six DNA sequences from three lines, represented by three colors. The genedropping part represents a cross between the blue and red lines and two generations of selfing. In the coalescence, the working unit is a current DNA stretch up to the most recent common ancestor (MRCA) for each pair of DNA sequences. The MRCA times are represented by t0,…t4. Once the coalescence graph is simulated, mutations (red arrows) accumulate in proportion to the length of each branch, note this means neutrality. In the Fig., the blue line is homozygous because no mutation has occurred between the two haplotypes. The coalescence does not directly allow for simulating complex traits and usually refers to small DNA fragments. In contrast, the forward process start with a base population of individuals, which would be the end point of the coalescence, and simulates forward matings. Phenotypes with any genetic basis can be simulated because each individual is generated in genedropping, whereas only the DNA sequences that left offspring are recorded in the coalescence. Because of that, all individuals at any time in point need to be kept in memory with genedropping, which is costly for many individuals and generations. Note the vertical scale in generations is very different between coalescence and genedropping

The traditional approach to simulate genomes is symbolic in nature: a computer program prescribes rules of how alleles segregate, how individuals mate (at random or not), the generation structure of the population, and selection rules. Here, there are two main approaches: the coalescence, or upward simulation, and gene dropping, or forward simulation (Fig. 2). The coalescence (Hudson 1983; Nordborg and Tavare 2002) detaches the mutational and phylogeny processes. This abstract sentence simply means that all polymorphisms are considered as neutral, since the number of offspring per individual is treated as independent of its genotype. Therefore, the coalescence is useful for understanding demographic events such as bottlenecks or crosses between populations but is not appropriate for simulating selection. There are approximations that deal with non-neutrality in the colaescence (Hudson and Kaplan 1988) but they are computer intensive and not well suited for the kind of short-term problems posed by plant breeding. Besides, coalescence simulations slows down considerably when recombination events increase and require approximations for complete genomes (Chen et al. 2009). The coalescence is widely used in evolutionary studies but not so much in plant breeding, where the time window of interest is very short and the mutational process can then be neglected. This means that observed selection response in domestic plants is primarily drived by standing segregating variants and not by very recent mutations.

Gene dropping (Crow and Kimura 1970; Lacy 1989) in turn start with a given population to simulate offspring genotypes and their phenotypes. As such, this procedure is completely flexible and can easily incorporate any breeding decision, genotype by environment interaction, gene editing, etc. Gene dropping has a long tradition in the field and numerous softwares are available (e.g., Pérez-Enciso et al. 2017; Chen et al. 2022; Shrote and Thompson 2024; Bančič et al. 2025). So far, most applications involve one or a few quantitative traits. A main reason is the difficulty in specifying so many parameters, such as genetic correlations, realistically.

The main drawbacks of gene dropping are the large memory and computing times usually involved. The memory demand can be dramatically reduced if the positions of the crossover events instead of the whole set of markers are stored (Pérez-Enciso et al. 2000). Besides, gene droppping simulations are difficult to parallelize, except for some steps like individual simulation within generation or meiosis across chromosomes. To our knowledge, there is no breeding simulation software that takes advantage of specialized hardware such as GPUs.

As mentioned, coalescence relies on uncoupling the mutational and genealogical trajectories, i.e., neutrality is assumed, which is just what breeding is NOT about. However, a hybrid procedure was proposed by Pérez-Enciso and Legarra (2016), which consists of using coalescence simulated data as input for gene dropping. This option is implemented in popular software AlphaSimR (Chris Gaynor et al. 2021). It has the advantage that recent selection procedures can be easily simulated, while also accounting for ancient demographic effects. Using extant real data as input for the forward simulation is an additional appealing alternative, e.g., Pérez-Enciso et al. (2017). This latter strategy ensures realistic variability and linkage disequilibrium patterns, which are difficult to simulate genome-wide. Given the wealth of genomic data at the population level currently available, it is our recommended approach.

In summary, symbolic simulation is model based and highly parameterized. It is then particularly suited for well-defined problems where a realistic set of hyperparameter values, e.g., heritabilities or genetic correlations, can be specified.

## A new paradigm: generative AI

Many of us have been surprised by realistic face images that have been computer generated (e.g., https://generated.photos/faces) or by programs that change the sex or age of a real image. This technology relies on generative AI, whose goal is to create new data instances that resemble the original dataset. These data are usually dubbed ‘synthetic data’ as compared to simulated data. While standard simulation usually focus on a single or a few traits, typically normally distributed, genAI can potentially reproduce any arbitrarily complex phenotypic pattern such as fruit images or plant architecture.

In the genAI paradigm, and in contrast to symbolic simulation, no explicit relationship between genome and phenome is needed and there is no restriction on the number of traits or distribution assumptions. The main requisite of genAI is the availability of enough training data from where the patterns needed to generate new data can be learned, rather than the complexity of phenotype distributions or biological knowledge.

Historically, the lack of enough labeled data for training machine learning models was one of the main drivers for developing generative algorithms. These algorithms allowed to increase training datasets, which should be as realistic as possible to be useful. The oldest generative models are, allegedly, Hidden Markov Models and Gaussian Mixture Models, proposed early in the 1950’s. These methods are also employed in Statistics and their parameters are interpretable. In early days, generative and symbolic simulation approaches were similar. Later developments, though, opened radically different ways for genAI tasks. Generative modeling, as we know it today, started with two revolutionary approaches: Generative Adversarial Networks (GANs, Goodfellow et al. (2014) and Variational Autoencoders (VAEs, Kingma & Welling 2013). More recently, flow based and diffusion tools have become popular. Diffusion is utilized in well-known tools like DALL.E 2 (https://openai.com/index/dall-e-2/), which generates high quality images from text prompts, to fill damaged or missing parts of an image, among many other applications. Flow-based methods have the appealing property that the exact likelihood can be computed. Other kind of methods, autoregressive models, are appropriate for longitudinal data and have been implemented in ChatGPT and other text generating tools.

Generative models, as most machine learning applications, need a loss function to be minimized during training. A popular loss function in genAI is the Kullback–Leibler (KL) divergence. This function measures how much a model probability distribution *q(x),* say the distribution of synthetic data, is different from a true probability distribution *p(x),* the observed data:

$$KL(p|q) = \smallint p\left( x \right)\log \frac{p\left( x \right)}{{q\left( x \right)}}dx$$

KL divergence measures the information loss when *q(x)* approximates *p(x)*, providing a measure of the discrepancy between probability distributions across the entire parameter space. KL supports well-behaved optimization—an essential quality for generative AI models. It is particularly effective in high-dimensional settings, where other divergence measures may struggle to scale. Other popular metrics are the Jensen–Shannon distance and Wasserstein GAN (WGAN). Some loss functions in the genAI context are discussed in Supplementary file 1.

## Overview of generative algorithms

Autoregressive models (ARMs), as the name suggests, generate data sequentially such that each item probability is based upon previous events. ARMs are especially suited for longitudinal data, such as plant growth or climatic data. In the case of images, for instance, pixels are treated as ordered row wise or column wise. The distribution of the observed dataset **x** is.

$$p\left( {\varvec{x}} \right) = \mathop \prod \limits_{i}^{n} p{(}x_{i} {|}{\varvec{x}}_{j < i} ),$$

Where subscript refers to each dimension of the data. This model is completely general and flexible but requires an enormous number of parameters as dimensionality grows. To make solutions tractable yet useful, numerous simplifications that force a constraint on the solutions are available, NADE (Larochelle and Murray 2011), PixelRNN (Oord et al. 2016) are among myriads of available ARR algorithms.

Performance of ARR models differ depending on algorithm and application. They excel at sequential data such as weather forecasting (e.g., Chin and Lloyd 2024; Kim et al. 2024). Recent ARMs based on transformers have revolutionized text generation and other generative applications, ChatGPT is a well-known example. A limitation for interpretability of plain ARMs is that they do not directly learn unsupervised representations of the data. They are designed primarily for next-token prediction —such as generating words in text—and therefore they are less effective at feature extraction. However, modified architectures incorporating attention mechanisms (Vaswani et al. 2017) may help address this limitation.

Generative Adversarial Networks (GANs, Goodfellow et al. 2014) are some of the most fascinating algorithms proposed in the AI domain. GANs are composed of two networks, a generative network that produce synthetic samples and a discriminative network that labels each sample as synthetic or real. Both are trained with opposing targets: while the generative network aims to maximize the verisimilitude of the output, the goal of the discriminative network is to correctly classify data instances. The equilibrium is reached when real and synthetic data are indistinguishable and so the discriminator fails 50% of the times, i.e., when data are classified at random. This is a minimax game because is a zero-sum game and minimizes maximum loss. As opposed to other methods described here, such as flow-based methods, GANs do not require the map function to be invertible and are not likelihood based, rather a latent space is used. GANs enjoyed immediate success and literally thousands of manuscripts have been published on the topic, over 20,000 papers as of year 2021 (Joshi et al. 2021). Applications in Agriculture are equally numerous (Lu et al. 2022).

For our purposes, the most relevant aspect is how the generator works. As in other generative models, the generative network learns to map from a latent space to a data distribution of interest. This latent space is initialized to random numbers but, after training, it acquires some ‘meaning’. This process is called ‘representation learning’ and makes the latent space interpretable (Bengio et al. 2012). The trick is that this interpretability needs to be discovered. For instance, a given latent space axis can be associated with sex or age in human face images (Radford et al. 2015). When the coordinate value is moved along the axis, images of males or females or of different ages are generated, with intermediate images in between. In our setting, for example, a GAN trained on fruits at various maturity stages could reveal a latent space corresponding to maturity. This would enable breeders to predict how fruits from different genotypes will develop over time.

Another potentially interesting application of GANs in breeding is the so called CycleGAN (Zhu et al. 2017). CycleGAN allows to transfer styles between images, e.g., a photograph can look as an impressionist painting and vice versa. This is done by having two linked GANs that generate paintings and photographs, and matching between them is ensured by adding an error term that measures discrepancy within actual pairs. In Agriculture, this has numerous potential applications, e.g., to ascertain how infested and healthy leaves would look like across species or different parts of the plant.

Despite its popularity, GANs have some well-known problems, such as training instability and being prone to mode collapse, i.e., they tend to generate samples from a reduced space only (Bishop and Bishop 2023). As a result, GANs may not be the best choice compared to the other methods described here.

Variational Auto Encoders (VAEs, Kingma and Welling 2013) rely on the concept of autoencoder (AE), a deep learning architecture where input and output are identical. A Variational Autoencoder (VAE) is a probabilistic extension of an autoencoder that learns a latent space distribution rather than a fixed encoding. Instead of mapping inputs to a single point in latent space, it maps them to a probability distribution that allows generating new data by sampling from this distribution. In order to return non-trivial solutions, autoencoders impose constraints on the learned representations. Principal component analysis (PCA) is actually a particular case of autoencoder where linear activation functions and a single layer network are assumed (Bishop and Bishop 2023). The AE solution is different from PCA though when several layers and nonlinear activation functions are used.

A VAE consists of an encoder that maps input ***x*** into a probabilistic latent space with prior typically chosen as *p(z)* ~ N(0,1), and a decoder that reconstructs the original data. The aim is to approximate a true posterior distribution *p(z|x)* using a distribution *q(z|x)* = N(µ*,σ*^2^) where µ and σ are learned by the network minimizing KL divergence between *p(z|x)* and *q(z|x).* In contrast to standard autoencoders, which result in a defined vector in the latent space, VAEs return a probabilistic distribution. Then, once the model is trained, new observations are generated by sampling from this distribution *q*(.) and running the decoder network. An advantage of VAEs, shared with other genAI methods, is that a latent space is learnt, which can simplify interpretation. Following previous reasoning, PCA can be thought of as a latent space where the weights of each variable help interpreting the components (Bishop and Bishop 2023). VAEs, as GANs, have been widely used in Agriculture for generating fruit shapes, (Zingaretti et al. 2021; Akkem et al. 2024) and references therein.

Difussion models (Sohl-Dickstein et al. 2015; Cao et al. 2022) consist of two parts, as VAEs, but their mechanism is completely different. First, random noise is gradually added to the input in successive steps so that it becomes completely blurred, the forward diffusion process, and second, a neural network is trained to remove the noise and recover the original data, the reverse diffusion process. The whole process is a Markov chain. At i-th step:

$$q(x_{i} | x_{i - 1} ) = N\left( {\sqrt {1 - \sigma_{i}^{2} } x_{i - 1} , \sigma_{i}^{2} } \right),$$

where *N* refers to the normal distribution, and ${\sigma }_{i}^{2}$ is the variance at the i-th step, which is a small number increased in each step. Collapsing all steps we have

$$q(x_{t} | x_{0} ) = N\left( { \sqrt {\overline{\alpha }_{t} } x_{0} , \left( {1 - \overline{\alpha }_{t} } \right){\mathbf{I}}} \right),$$

where ${\overline{\alpha }}_{t}= {\prod }_{i=1}^{T}(1- {\sigma }_{i}^{2})$ and $q({x}_{t} | {x}_{0} ) \to N(\text{0,1}).$ During training, the algorithm learns the reverse functions ${p}_{\theta }({x}_{i-1} | {x}_{i})$ using neural networks with parameters *θ* and KL divergence as loss. These functions are also normal distributions with parameters depending on observed *x*_*i*_. A generative process logically follows: start with a newly generated random noise and run the trained denoising network. Diffusion can be quite computer intensive since it involves running a denoising process with many, e.g., thousands of steps (Bishop and Bishop 2023).

Compared to VAEs and GANs, diffusion models usually produce less noisy output and still are very stable during training, although they can be slow. They have become widely used recently, and become state of the art in some popular applications like DALL-E 2 (Ramesh et al. 2022), which generates images out of text prompts. In breeding, an equivalent application of text-to-image tools would be to generate plant features, e.g., fruit shape, out of DNA sequence (Pérez-Enciso et al. 2022; Jurado-Ruiz et al. 2023). These authors used autoencoder based models though. Diffusion models have become recently popular in Agriculture. There exist numerous applications in this area, mostly for image analysis such as segmentation (Heschl et al. 2024), weed and disease identification (Muhammad et al. 2023; Chen et al. 2024; Mori et al. 2024; Zhou et al. 2025), or augmenting fruit datasets (Zhao et al. 2024), to name a few.

**Fig. 3:**
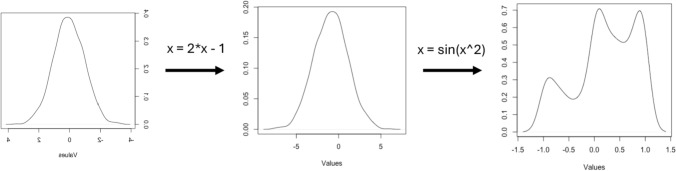
Successive functions applied to an initial simple distribution can fit complex data patterns. The Fig. shows density plots of a N(0,1), followed by an affine transformation and a sinus of squared value

Flow-based generative models were proposed by Dinh et al., (2014, 2016) and (Kingma and Dhariwal 2018) among others. Flow methods are able to compute likelihood exactly yet sampling is efficient. The key observation is that a simple probability function can be converted into another one of arbitrary complexity using change of variable probability methods (Fig. 3). The change in variable steps should be invertible functions such that the whole process can be reversed without loss of information, which is called a normalizing flow (Rezende and Mohamed 2015; Kobyzev et al. 2019). Take *z*_*0*_ ~ *p*_*0*_ = N(0,1), *z*_*1*_ = *f*_*1*_*(z*_*1-1*_*), …*,* z*_*i*_ = *f*_*i*_*(z*_*i-1*_*)* where *f*_*i*_ are invertible functions. The log—likelihood after *K* transformations is

$$\log \left[ {p_{k} \left( {z_{k} } \right)} \right] = \log \left[ {p_{0} \left( {z_{0} } \right)} \right] - \mathop \sum \limits_{i}^{K} \log \left| {\det \frac{{\partial f_{i} \left( {z_{i - 1} } \right)}}{{\partial z_{i - 1} }}} \right| ,$$

(Bishop and Bishop 2023), where |.| refers to the absolute value and *det(.)* are determinants of the Jacobians. Using this, an interesting property of the flow approach is that the likelihood is explicitly modeled and learnt. Each transformation function *f*_*i*_ is typically parameterized by a neural network, which are trained to minimize KL divergence using standard methods**.** These networks must be designed to ensure **i**nvertibility. Fully connected layers or convolutional neural networks are not suited for this case, some examples are RealNVP (Dinh et al. 2016) or NICE (Dinh et al. 2014) algorithms. Sampling in flow generative models is very efficient.

**Fig. 4:**
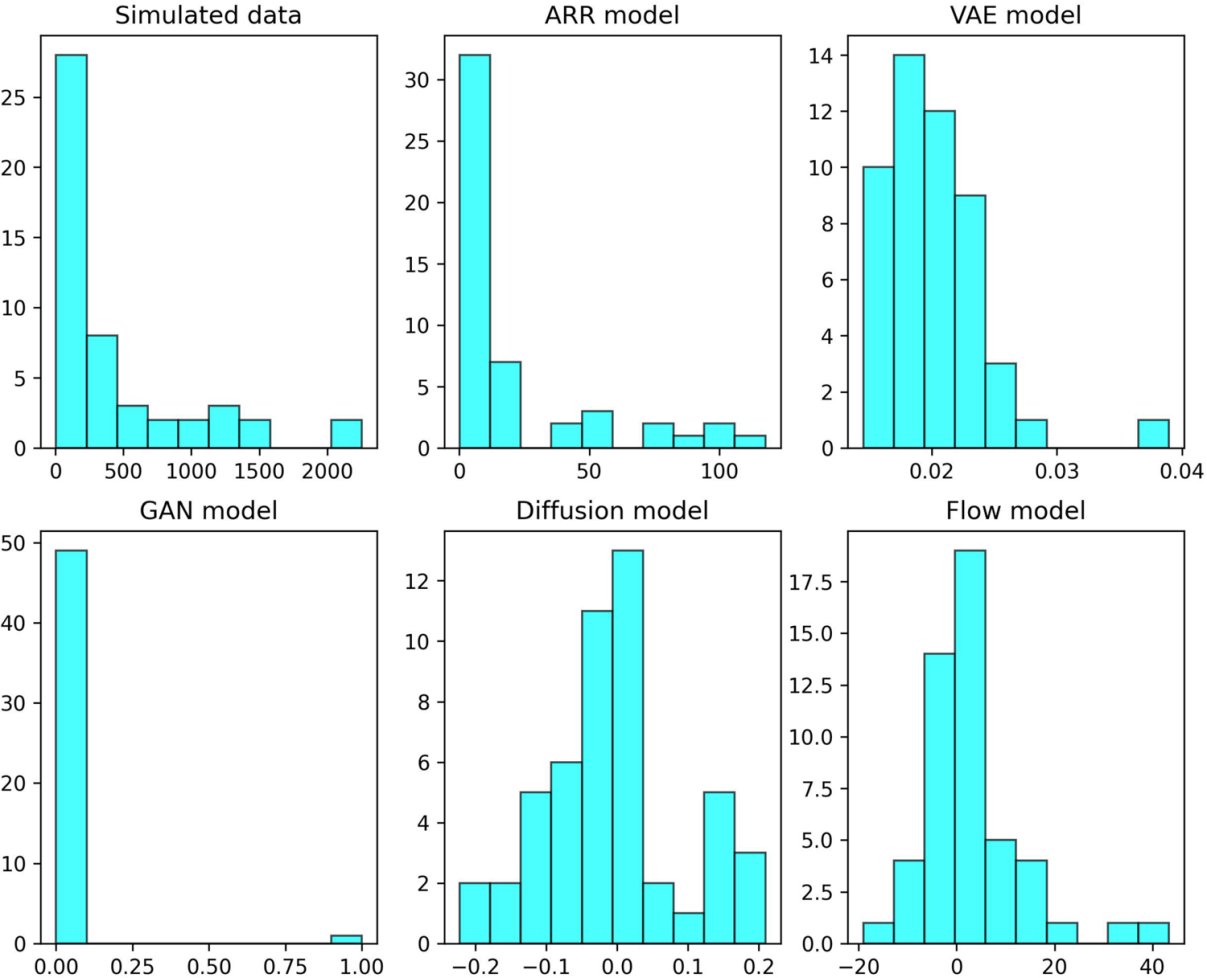
Histograms of simulated microbiome abundances and of synthetic data. Simulated dataset comprises 1,000 samples and 50 species. Abscissas are species mean abundances and ordinates, species counts. Code generated by ChatGPT without fine tuning

A comparison of the five generative models applied to microbiome simulated data is in https://github.com/miguelperezenciso/genAI. The Pytorch code was generated with ChatGPT and manually checked and polished but no fine tuning was attempted. Therefore, the code provided is intended only to visualize the algorithm structure. Nevertheless, some interesting issues can be observed (Fig. 4). First, the performance of each model vary largely and not every algorithm will be appropriate for the type of data at hand. Here, we observed that the vanilla autoregressive model performed best, whereas the VAE and the diffusion algorithms did rather poorly. Note that a big improvement could be achieved if flow output would be the absolute value instead of the signed number. A second issue worth noticing is that GANs collapsed and generated only two abundances, either 0 or 1, a behavior often observed with these algorithms.

**Fig. 5:**
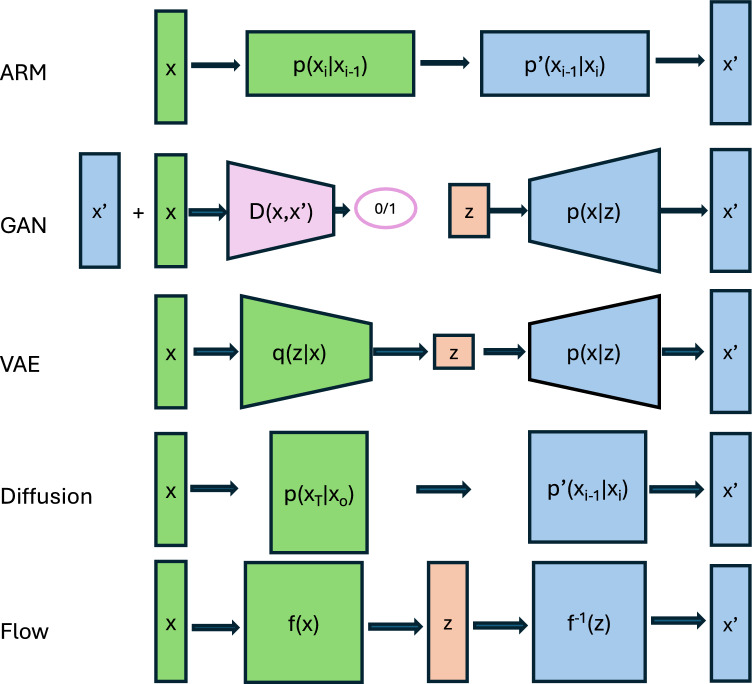
A cartoon representation of discussed genAI algorithms. Green and blue rectangles contain observed (**x**) and synthetic (**x’**) data; all genAI algorithms contain a forward (green) and backward (blue) parts, except GANs, that contain a discriminator (magenta). The latent space **z**, if explicitly defined, is in orange rectangles. The varying dimension of the rectangle **z** represents grossly the dimensionality of the latent space, smaller in VAEs than in flow and diffusion methods. Note the similarity in structure between diffusion and autoregressive models, with some differences: ARMs model the data themselves whereas data are successively blurred in diffusion; these can be obtained directly in one step, p(x_T_|x_o_), whereas that is not possible in ARMs. In diffusion, the blurred data might be considered kind of latent space, but that is not the case in ARM. Modified from L. Weng blog (https://lilianweng.github.io/posts/2018-10-13-flow-models/)

Table 1 summarizes the main features of each method as inferred from the literature, and Fig. 5 compares them graphically. Historically, GANs and VAEs have been very popular, although ChatGPT, an autoregressive technique, and DALL-E 2, a diffusion model, have recently enjoyed great success. To our knowledge, flow modeling has not been applied yet to predictive breeding but is worth exploring due to speed and quality of data generated. Despite their attractive properties, flow-based models have not been widely in Agriculture, with a few exceptions such as plant disease detection (Kim et al. 2022) and remote sensing (Irigireddy and Bandaru 2025). A limitation of flow-based methods is that the latent space is not of reduced dimensionality; therefore, this approach may not provide an interpretable representation of the data as other techniques such as VAEs may do. In turn, VAEs have a low dimension latent space but generates images of low quality. Each type of data may require a different approach and is difficult to provide general recommendation of which one may be more appropriate (Fig. 4).

**Table 1:** Comparison between generative models

Method	Description	Training stability	Sample quality	Collapse risk	Computational cost	Interpretability	Good for
Autoregressive	Generate data sequentially	Good	Good	Low	High	Poor	Longitudinal data
GAN	A generator and a discriminator network compete	Poor	Very good	High	Low	Fair	Images
VAE	Encode data into a probabilistic latent space	Good	Poor	Low	Low	Good	Representation learning, style transfer
Diffusion	Generate data reversing a gradual noising process	Very good	Very good	Low	High	Poor	Images, audio
Flow	Generate data by applying invertible transformations	Very good	Good	Low	Low	Fair	Images

## Hopes for generative AI

Generative AI is under active development and is, potentially, a disruptive technology for Agriculture. It is also a big business, with capacity to influence all aspects of economic activity (Precedence Research 2024; Salari et al. 2025). The ability of these algorithms to generate data of very high quality, in particular images, is really surprising. Reliability and versatility of synthetic data can only increase in the coming years. The availability of pretrained algorithms on large amounts of data should allow smaller farmers and breeding companies to leverage this technology and adapt it to their needs.

As mentioned above, generating realistic phenotypes in simulation studies is fundamental and can explain differences between simulated and observed results. Traditional simulations tend to be overoptimistic, partly because evaluation models assume rather simplistic phenotype – genotype maps. A major advantage of genAI is the possibility of generating numerous data instances of extreme realism without specifying model parameters. This might allow the breeder, e.g., to explore the expected phenotypic variability in the germplasm available. For instance, a GAN trained with tomato pictures from pure lines can deliver expected images from all potential crosses together with their variability within cross. phenotypic variability conditional on parents’ (Pérez-Enciso et al. 2022). No doubt, many more applications of genAI will be developed in the near future.

Transformers (Vaswani et al. 2017) have revolutionized AI, in particular for analyzing massive amounts of text, but also video or images among other applications. GenAI has leveraged this new architecture that allows to process massive amounts of data in parallel and find connections between distant items. As an example, some genomic language models (gLM, Benegas et al. 2025) use transformer algorithms to analyze DNA data, which, like language, are characterized by their sequential nature and the potential significance of distant connections (Ji et al. 2021). These models have a big potential in genomics and, therefore, in breeding. The generation of completely new genomes with genomic language models might be possible in the future (Benegas et al. 2025). Therefore, the genotype simulator represented by module G2G (Fig. 1) could eventually be a generative DNA language model. Their properties, however, are unknown since Mendel laws and recombination distances will likely not be used. It remains to be seen whether those synthetic genomes could actually exist.

## Caveats

There are three well known drawbacks of genAI: transferability, explainability and the presence of ‘hallucinations’. Explainability is built into standard simulation: input parameters have well known characteristics and their impact can be assessed. In contrast, broad explainable AI remains a controverted idea and a desideratum rather than an accomplished goal (Angelov et al. 2021).

Transferability refers to the capability of AI algorithms of returning sensible outputs when previously unseen datasets are employed as input. GenAI only learn patterns from previously observed datasets. They cannot generalize to any new scenario. For instance, it is dubious that genAI can predict the effect of any new mutation in a genome, at least with current data available. The ability to extrapolate of current genAI algorithms is limited by the dataset on which genAI is trained.

‘Hallucinations’ are unrealistic or non-sensical generated items (Rawte et al. 2023). In a standard statistical context, they would be somewhat equivalent to ‘outliers’, and would be typically removed from the analyses. In the realm of text generative tools like ChatGPT or DeepSeek, hallucinations are misleading and can be quite dangerous. This danger is not so evident in the scenario studied here. However, genAI will likely produce data that may not look outliers but might be impossible to observe, especially in highly dimensional data. An example could be a DNA sequence with a large amount of recombinants or an impossible combination of phenotypic values. The true impact and the frequency of hallucinations in the context of this work remains to be studied. To achieve this, plant biology and breeding expertise, along with AI knowledge, are needed.

Note genAI will improve the quality of data generated but will not necessarily improve our understanding of agronomic processes or discover underlying rules of Biology. Added to this, genAI models are highly parameterized and can result in ‘equifinality’, *i.e.*, different models may return similar data, complicating interpretability.

Although there is extensive experience of genAI in image and text generation, its performance in agronomic traits is less well known. For instance, to our knowledge, there are no applications of genAI to plant growth data and yield across locations in the presence of genotype $\times$ environment interaction. GenAI is supported by rigorous theory but implementation details and hyperparameter optimization are mostly driven by trial and error. The lack of pretrained models can be problematic, since adjusting genAI architecture and hyperparameters is time consuming and, sometimes, frustrating. Large public datasets, such as genomes to fields in corn (Lawrence-Dill et al. 2019), could be used to train genAI algorithms. This issue is worsened by the lack of user-friendly software. Admittedly, simulation software is not necessarily ‘friendly’ either but requires less expertise in the domain. A final consideration regarding genAI is the need of large computer resources and large datasets for training.

## Will generative AI supersede traditional genetic simulations?

Computer symbolic simulation has been around for decades and few technological breakthroughs can be expected. Generative tools, in turn, are such an active area of research and so powerful that it is legitimate to ask whether they will eventually replace traditional (symbolic) simulation methods. In our opinion, the use of genAI can only grow in the coming years but is unlikely that it will replace symbolic simulation. Often, simulations of enough complexity can be programed in a few lines, e.g., with specialized libraries in R programming language. So far, that is not possible with genAI, although this may change in the future.

**Fig. 6:**
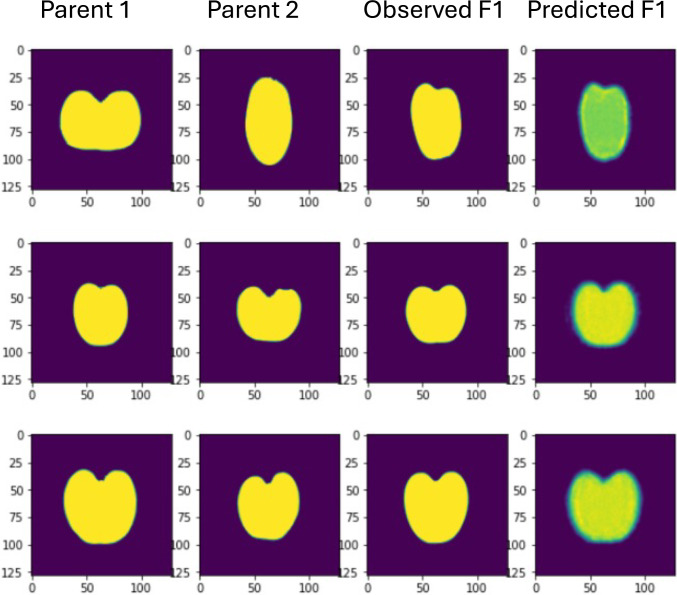
Shape profiles of three tomato crosses with their observed and predicted shapes. Predictions are based on an encoder – decoder architecture where the encoder input is made up of the two parental images. Training was carried out removing the shown crosses. Redrawn from Pérez-Enciso et al. (2022)

An interesting application of genAI would be phenomic selection, which consists of selecting individuals based on highly dimensional phenotypes, such as spectroscopy bands (Rincent et al. 2018). In this scenario, genAI could be used to generate performance of offspring based on phenotypes only. For instance, Pérez-Enciso et al. (2022) showed that fruit shapes of offspring could be produced out of parentals’ shapes in tomato and that genic action was primarily additive (Fig. 6). In this case, authors used an autoencoder architecture.

Symbolic simulation is often used for studying the effects of varying well-defined parameters (generation interval, heritabilities, …). In this sense, lack of interpretability in genAI will remain a hindrance compared to standard simulation, even if latent spaces can be useful in this respect. Representation learning is ‘learning representations of the data that make it easier to extract useful information when building classifiers or other predictors’ (Bengio et al. 2012). Latent spaces are the main tool for this task but latent spaces per se are not interpretable, additional analyses are needed to discover the impact of each dimension of the latent space.

In all, we argue that augmenting data as realistically as possible will improve inference and predictive performance of genomic prediction models, but parametric simulation will continue to play a fundamental role in predictive breeding.

## Can we get the best of both worlds?

We have shown that, while symbolic simulation and generative AI use very different methods, they also share similarities and can serve similar purposes (Fig. 1). In particular, both are able to generate new, realistic data. Most research on genAI so far has focused on phenotypes, but the same principles can be applied to generate genotypes or synthetic environments. The main difference between traditional simulation and genAI lies in how the data are generated. Simulation is a model-based process, whereas generative AI operates through imitation. GenAI can be quite powerful to produce synthetic phenotypes, genotypes and environments but symbolic simulation provides the tools to combine these data, e.g., mating strategies or physiological mechanisms such as CGMs (Technow et al. 2015; Messina et al. 2018). Can we envisage a procedure that combine both advantages?

The holy grial of breeding is to predict the performance of genotypes for traits of interest across all possible environments on which the genotype might be grown in the future. In the case of hybrid crops, prediction is extended to any potential cross that might be produced. With this purpose, genAI could be used to simulate performances given genotype and environment features. For instance, De los Campos et al. (2020) presented a simulation approach in the spirit of generative modeling, whereby they used extensive trial data to train a model that learns patterns of how genotypes and environmental conditions influence phenotypes, and then used the trained model to simulate performances by prompting it using historical weather data. Another point of connection of the approach presented in this study and generative AI is that the model **Y** ~ *f*(**G, E**) was not fixed, they also incorporated uncertainty about model parameters in the process of simulating data.

To use genAI within the GE2Y module (Fig. 1), generative algorithms *conditional* on genotype and environment features are a natural choice. Simulated genotypes could be obtained with standard classical algorithms, or with genomic language models in the future (Benegas et al. 2025). The main advantage of this proposal is that we do not need to specify a map linking genotype and phenotype, since it would be learnt from the data via the latent space, but we still retain all advantages of genAI such as dealing with any number of realistic phenotypes of any complexity. The disadvantage is that large datasets are likely needed and that results may not be transferable to other input datasets. This requisite can be alleviated using pretrained models adjusted via transfer learning.

The following steps, once phenotypes have been obtained, would be identical to a standard simulation software, e.g., breeding values can be predicted using, e.g., GBLUP, or with predictive AI tools such as convolutional neural networks. Further, the whole breeding scheme could be optimized with reinforcement learning to complete the whole algorithm (Moeinizade et al. 2022; Younis et al. 2024).

The single most relevant feature in our proposal is ‘conditional’. While genAI developers focus on getting data as realistic as possible, breeders are interested in the performance of given genotypes in specific (or even unknown) environmental conditions. Note that, in the genAI framework, we do not need to assign a particular effect to a polymorphism in the genome. Rather, the conditional generative algorithm will empirically learn the map genotype to phenotype. It remains to be studied the behavior of conditional genAI in this context but it can be conjectured that very large datasets might be needed.

**Fig. 7:**
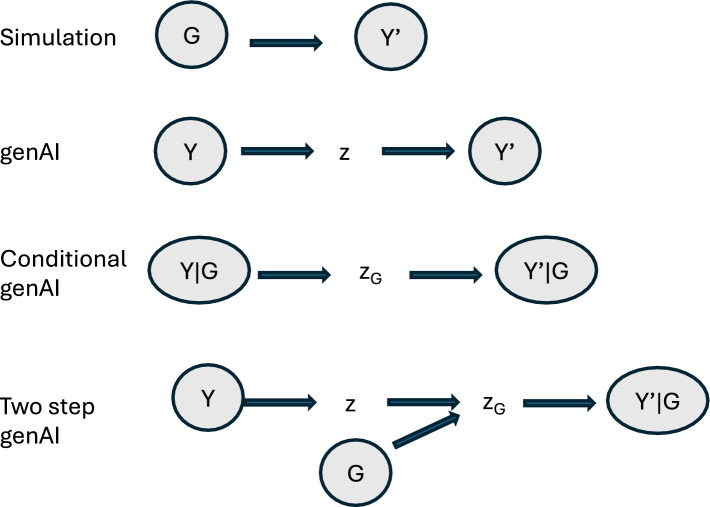
The four options to generate phenotypic data discussed here. Environmental effects are not shown for conciseness. Symbolic simulation uses a well-defined function to obtain new phenotypes (**Y’**) from genotypes (**G**). Generative AI generates **Y’** starting with a current phenotype dataset (**Y**), often using an intermediate latent space (**z**). The most straightforward way to use genAI in a simulation context is to generate **Y’** conditional on **G** (and environment). The problem with this approach is that the number of levels in **G** is very large compared to standard problems in conditional genAI. A way to circumvent this is to assign a genetic basis to a low dimensional latent space, e.g., sample **z**_**G**_ ~ N(**0**, **A**) where **A** is the genomic relationship matrix and run the trained decoder

As mentioned, latent spaces are internal representations of the input data which are usually of lower dimension than the original input and can represent salient features of the data (Bengio et al. 2012; Bishop and Bishop 2023). Latent spaces provide a link between simulation and genAI. One option is to assign a genetic basis to the latent space, treating the latent space as an intermediate phenotype, which could be simulated using standard software. Then, the trained network could generate new observations using these simulated latent space values. This approach may not be applicable to all generative algorithms. GANs, VAEs, flow-based and some variants of diffusion models use latent spaces whereas autoregressive models do not. Dimensionality of latent spaces vary, those of flow models are of the same dimensionality as the raw data and so are not appropriate for this application. We conjecture that VAEs or similar genAI algorithms are better suited. A cartoon of our proposal compared to other approaches is in Fig. 7.

## Discussion and future directions

Standard simulation has long played, and will continue to play, an invaluable role in predictive breeding but few breakthroughs can be foreseen. Generative AI, in contrast, is a very active research area and new algorithms emerge constantly. Here, we reviewed some of the most relevant ones (Table 1, Fig. 5). Modern generative algorithms are able to produce synthetic data, such as images, of extremely good quality. As opposed to highly parameterized simulation tools, genAI makes no underlying assumption on the phenotype distribution. This property is certainly attractive for highly multidimensional traits, e.g., fruit shapes or plant architecture across developmental stages. However, it is unlikely that generative models of the kind Y2Y (Fig. 1) are useful per se in breeding unless a genetic component is considered, and several options have been discussed here.

A shared principle of most genAI algorithms is that they rely on the concept of ‘latent space’. We argued that one option would be to assign a genetic basis to the latent space, which would allow simulating the genetic transmission of highly complex phenotypes. Within this framework, a VAE derived method looks optimal due to the low dimensionality of the generative latent space, simply a normal. A disadvantage is that VAEs output is not of very high quality, and so hybrid strategies like latent diffusion models or latent flow-based models (Xiao et al. 2019; Zhang et al. 2021; Rombach et al. 2021) may work better.

Revisiting Fig. 1 allows us to recapitulate and envisage future challenges. In terms of genAI, most of the work presented here revolves around module Y2Y, i.e., algorithms that produce new phenotypes by learning from extant data. In contrast to classical simulation strategies, genAI algorithms can reproduce multivariate distributions of arbitrary complexity. This feature per se, as mentioned, is not useful for breeding purposes unless a genetic component is incorporated. We have discussed two alternatives: the first one is to use conditional generative tools, where conditioning is on genotype and environment (module GE2Y), the second one is to model a genetic basis for the latent space (Fig. 7).

Finally, Fig. 1 includes two additional modules that we have barely mentioned: G2G and E2E. Again, these two modules can be implemented either symbolically or in a data—driven framework. Symbolic G2G comprises well known algorithms that generate offspring genotypes given parental genotypes and recombination rates. In contrast, data—driven G2G is much less studied. In the future, we can envisage DNA language models capable of synthetizing new genomes whose performance would be evaluated entirely in silico (Callaway 2025; Brixi et al. 2025). Environmental data can be simulated (E2E), the simplest approach is sampling from normal noise but sampling from observed past climatic conditions is an option (de los Campos et al. 2020). Alternatively new environments can be synthetized with genAI using observed weather and soil from public databases as input for learning.

## Supplementary Information

Below is the link to the electronic supplementary material.

Supplementary file1 (PDF 96 KB)

## Data Availability

ChatGPT generated code for all methods discussed and applied to simulated microbiome data are in https://github.com/miguelperezenciso/genAI.
