# Supplementary material for: Generative AI for predictive breeding: hopes and caveats
Source: Theor Appl Genet. 2025 Jun 11;138(7):147. doi: 10.1007/s00122-025-04942-8 (PMC12159116; doi:10.1007/s00122-025-04942-8)
Supplement: Supplementary file 1 — Supplementary file1 (PDF 96 KB) [file 122_2025_4942_MOESM1_ESM.pdf]

Loss functions in the context of generative AI

A popular loss function in genAI is the Kullback-Leibler (KL) divergence. This function measures how much a model probability distribution $q(x)$, say the distribution of synthetic data, is different from a true probability distribution $p(x)$, the observed data:

$$KL(p|q) = \int p(x) \, log \, \frac{p(x)}{q(x)} dx$$

KL divergence measures the information loss when $q(x)$ approximates $p(x)$. Originating from information theory, it is a combination of basic entropy formulas. The entropy of a probability distribution is

$$H(p) = -\int p(x) \, log \, p(x) dx,$$

indicating the minimum number of bits or nats (the lower bound) required to encode events drawn from $p(x)$. Cross-entropy between $p(x)$ and its approximation, $q(x)$ is expressed as follows:

$$H(p, q) = -\int p(x) \log q(x) \, dx,$$

$H(p,q)$ is a measure of the minum number of bits or nats required to encode outcomes from p using a code optimized for q, and $\mathrm{H}(p, q) \geq H(p)$. The KL divergence formula can be rewritten as follow:

$$KL(p|q) = \int p(x) \log p(x) \; dx - \int p(x) \log q(x) \; dx,$$

$$KL(p|q) = H(p, q) - H(p)$$

KL divergence provides a way to measure the discrepancy between probability distributions across the entire parameter space, supporting well-behaved optimization—an essential quality for generative AI models. It is particularly effective in high-dimensional settings, where other divergence measures may struggle to scale.

KL divergence is a core component of the loss functions in several key generative frameworks, including variational autoencoders (VAEs), diffusion models, and reinforcement learning-based approaches. Its tractability and compatibility with gradient-based optimization make it a powerful tool in training complex generative models.

Note that KL is a divergence and not a distance, as $KL(p|q) \neq KL(q|p)$. Indeed, $KL(q|p)$ is known as the reverse KL divergence, is also used as part of the loss function in various generative AI models, particularly in variational inference frameworks such as Variational Autoencoders (Kingma & Welling, 2014).

Another popular divergence function is the Jensen – Shannon, defined as

$$JSD = \frac{1}{2}(KL(p|m) + KL(m|p)), m = \frac{1}{2}(p(x) + q(x))$$

The intuition behind JSD is that it effectively averages the two asymmetric components of Kullback-Leibler (KL) divergence. The JSD is non-negative and becomes zero only if both distributions are identical. Unlike the KL divergence, however, it is symmetric with respect to the two distributions (Bishop and Bishop, 2023). In the original Generative Adversarial Network (GAN) formulation (Goodfellow et al., 2014), the training objective is based on minimizing the JSD divergence between the real data distribution, p_{data} and the generator distribution p_G. A comprehensive analysis of the relationship between Generative Adversarial Networks (GANs) and the JSD is available in Goodfellow et al., 2014.

Despite its theoretical elegance, the use of JSD in practice can lead to vanishing gradients, especially when the supports of p_{data} and p_G do not overlap, which is often observed in high-dimensional spaces. These limitations have motivated the development of alternative formulations. Wasserstein GAN (WGAN) replaces the JSD with the Wasserstein (or Earth Mover's) distance, which provides meaningful gradients even when distributions are disjoint, leading to more stable and robust training dynamics (Arjovsky et al., 2017). Furthermore, the f-GAN framework (Nowozin et al., 2016) generalizes the adversarial training objective by allowing the use of any f-divergence, including KL, reverse KL, Total Variation, and others, through a variational approach. This flexibility enables the training process to be tailored to specific modeling goals and data characteristics.

Divergence-based loss functions are crucial for training generative models by comparing real data with generated samples. JSD is fundamental to the original Generative Adversarial Network (GAN) framework, while the KL divergence—utilized in both forward and reverse forms—is frequently employed in models such as variational autoencoders (VAEs) and specific reinforcement learning scenarios. Due to the limitations of JSD and KL in cases of mode collapse, alternatives like the Wasserstein distance and other f-divergences have emerged. Each divergence measure presents theoretical and practical trade-offs that influence optimization stability, convergence behavior, and sample diversity. Thus, selecting an appropriate divergence measure is a critical design decision that significantly impacts the effectiveness and quality of generative models.

References

Arjovsky, M., Chintala, S., & Bottou, L. (2017). *Wasserstein GAN*. arXiv preprint arXiv:1701.07875.

Bishop CM, Bishop H (2023) Deep Learning: Foundations and Concepts. Springer International Publishing

Goodfellow IJ, Pouget-Abadie J, Mirza M, et al (2014) Generative Adversarial Networks. Sci Robot 3:2672–2680

Kingma DP, Welling M (2013) Auto-Encoding Variational Bayes. 2nd International Conference on Learning Representations, ICLR 2014 - Conference Track Proceedings. https://doi.org/10.61603/ceas.v2i1.33.

Nowozin, S., Cseke, B., & Tomioka, R. (2016). f-GAN: Training Generative Neural Samplers using Variational Divergence Minimization. arXiv preprint arXiv:1606.00709.
